# Development and Pilot Testing of the Tremor Retrainer Smartphone Application for the Treatment of Functional Tremor

**DOI:** 10.5334/tohm.823

**Published:** 2023-12-19

**Authors:** Jordan F. Garris, Gavin Bauman, Alberto J. Espay

**Affiliations:** 1 Department of Neurology, University of Virginia, US; 2 Bionic Panda, LLC, Georgia, US; 3 James J. and Joan A. Gardner Family Center for Parkinson’s disease and Movement Disorders, Department of Neurology, University of Cincinnati, US

**Keywords:** tremor, functional, entrainment, treatment, smartphone

## Abstract

**Background:**

Functional tremor is a common and disabling condition with limited treatment options. A prior proof-of-concept pilot study sought to translate entrainment, a key diagnostic feature of functional tremor, into a treatment strategy.

**Methods:**

The Tremor Retrainer smartphone application was developed though a collaboration between neurologists and a software engineer. It analyzes data from smartphone accelerometers to measure baseline tremor frequency, then provides auditory cues at a lower frequency for the patient to match with flexion-extension movements at the wrist. The application provides continuous biofeedback on performance via a visual gauge. Patients with functional tremor underwent a one-week treatment protocol with the Tremor Retrainer application and provided feedback on usability and acceptability to guide software programming.

**Results:**

Three pediatric patients completed the one-week protocol and their feedback was used to modify the software. All patients felt that the application was easy to use and could be effective in treating functional tremor.

**Discussion:**

The Tremor Retrainer smartphone application uses auditory cues and a visual gauge to provide a personalized and widely accessible entrainment-based intervention. Pilot testing in pediatric patients provided key feedback for application design.

**Highlights:**

The Tremor Retrainer smartphone application modulates functional tremor frequency by providing pulsed auditory cues for a patient to match with wrist flexion-extension movements while receiving continuous biofeedback via a visual gauge. This adaption of the diagnostic sign of entrainment has potential as an accessible treatment for patients with functional tremor.

## Introduction

Functional tremor is a common movement disorder associated with prolonged disability and limited treatment options [1,2,3,4]. It is distinguished from other movement disorders by such key diagnostic features as suppressibility and entrainment, whereby a patient’s attempts to match an externally cued frequency cause the tremor to abate or adopt this external frequency [5].

The feature of entrainment was adopted as a treatment strategy for 10 adult patients with functional tremor [6]. These patients underwent surface electromyography measurement of baseline tremor frequency. Patients were “cued” by tactile stimulation to adopt an oscillation 2/3 of the native tremor frequency, followed by 1/3 that frequency, while making flexion-extension movements at the wrist to match this externally cued frequency. Patients received continuous visual feedback on their performance and coaching as needed. If tremor was not dramatically improved after a single 2-hour session, patients returned for one or two further 2-hour sessions based on response. All patients improved following the entrainment sessions, with 60% of patients achieving long-term remission.

While this resource-intensive therapy is not available to most patients, the standard presence of accelerometers within modern smartphones makes this intervention readily adaptable for portable delivery. Therefore, we developed the Tremor Retrainer smartphone application for the treatment of patients with functional tremor, using patient experience and feedback to guide application development.

## Methods

The Tremor Retrainer application was developed through a collaboration between the authors, with concept conceived by movement disorders neurologist JG and software code written by GB, an experienced software engineer. The application is cross platform, running on iOS and Android using the Xamarin platform, and was written in C#. Development of the core tremor detection algorithm relied on device accelerometer parameters extracted in 3 axes (x, y, z) and analyzed via Fast Fourier Transformation to determine dominant baseline tremor frequency. Treatment frequency was defined as 2/3 of baseline tremor frequency for the first half of a session, then 1/3 of baseline tremor frequency for the second half of a session. A visual gauge was added with needle position determined by frequencies of user’s voluntary movements relative to goal treatment frequency. Auditory cues were added as a “ticking” sound matching treatment frequency. Video 1 demonstrates the use of the application by a healthy volunteer.

**Video 1:** **Application Demonstration**. Demonstration of user beginning a treatment session with the Tremor Retrainer application, including initial measurement of baseline oscillatory movement and biofeedback to reflect the user’s accuracy in reaching goal frequency of voluntary movement. After stopping a session, the user can view a log of duration completed.

**Figure 1:**
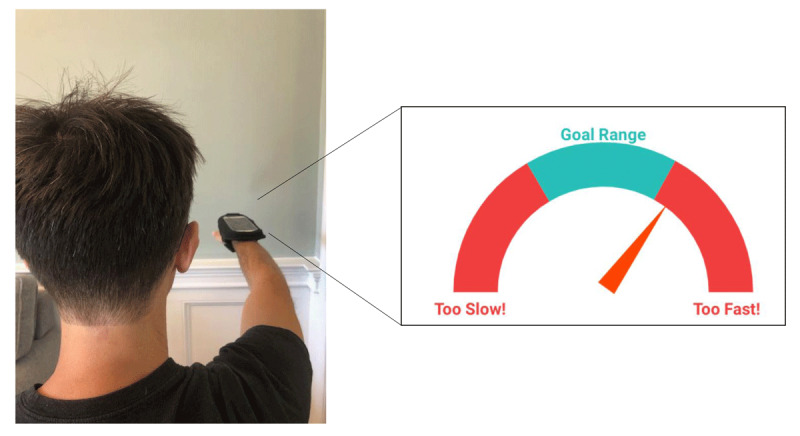
**Visual Gauge**. Users receive continuous biofeedback via a visual gauge throughout a treatment session. The needle moves in response to the frequency of the user’s voluntary movement: a patient matching the goal frequency will see a needle pointing to the center of the “goal range.”

For the seven-day treatment program, the Tremor Retrainer smartphone application allows users to select the treatment day (Day 1 = 60-minute session, Days 2–7 = 30-minute sessions) from the home page. An instruction page prompts the user to extend arms with the smartphone strapped to the wrist and then “match the beat” by moving the hand up and down at the wrist. After a session is initiated, the application computes the average baseline tremor frequency from the first 10 seconds of a session. Following this baseline measurement period, the application provides auditory cues at 2/3 of the baseline tremor frequency for half a session, then 1/3 of the baseline tremor frequency for the second half of the session, while continuously measuring the frequency of the patient’s adopted (voluntary) flexion-extension wrist movements as they attempt to “match” the cued frequency. Measured frequency of voluntary movements is represented on a visual gauge, with goal range in the middle (Figure 1). A countdown timer displays the remaining duration of a session. Once a session is completed or ended by the user, the user can view a time-stamped log of duration completed.

The UVA Institutional Review Board (HSR220215) approved the recruitment of both pediatric and adult functional tremor patients for feedback on application development. Written informed consent and assent was obtained from all subjects and their guardians. Subjects received instruction from the primary investigator on application use and were observed completing a 60-minute session in the clinic. Subjects then were instructed to complete 30-minute sessions on days 2–7 at home. Subjects underwent structured interview after the initial session and after the entire protocol regarding usability of the application. Patients were asked about technical difficulties they encountered, any fatigue or soreness, whether they felt the application was easy to use, whether sessions were reasonable to complete at home, and whether they felt the application could be effective in treating their functional tremor. Separately, patients and their guardians were asked to report the impression of change in tremor severity using the patient-rated Clinical Global Impression-Improvement (CGI-I) scale.

## Results

Three girls (age 17, 13, and 17) with functional tremor completed the one-week Tremor Retrainer protocol using Samsung, Motorola, and iPhone smartphones. No adult patients were referred for study participation during the study duration. All three subjects completed 100% of the initial 60-minute session. Subjects 1–3 completed 100%, 51%, and 83% of at-home sessions, respectively. Reasons for not initiating at-home sessions included inadequate time and psychological distress resulting in not wanting anything to touch the body. Reasons for early termination of sessions included application crashing and arm fatigue.

Observation of the initial session and feedback from patients prompted further software adjustments to address software bugs and improve the user experience. These included 1) creating minimum and maximum treatment frequencies to account for absence of detectable tremor when starting a session and to improve tolerability of sessions; 2) adjusting the code to ensure the visual gauge needle is centered in the goal range when patients are attempting to accurately match the new frequency; and 3) adjusting the countdown timer location to improve visibility.

During the structured interview, two subjects reported mild transient arm soreness and fatigue that resolved within minutes after a treatment session. All three subjects indicated that the application was easy to use, that the protocol was reasonable to do at home and could be effective in treating their functional tremor. The three subjects reported minimal improvement in tremor severity by CGI-I. Guardians also completed the CGI-I scale for 2 subjects: guardian of subject 2 noted marked improvement while guardian of subject 3 noted no change.

## Discussion

We present the development of the Tremor Retrainer smartphone application for delivering biofeedback-based treatment to patients with functional tremor, with patient feedback contributing to improving key features. The application measures and modulates functional tremor frequency, with auditory cues and a visual gauge to guide the user. Patient feedback during the development prompted modifications in response to patient-specific factors.

In our small sample, three teenage patients with functional tremor were able to complete most sessions, including at home, with psychological distress the most common reason for inability to participate in sessions. This is an important preliminary indicator of feasibility as the prior entrainment study involved only adult patients with in-person coaching. This study involved for the first time pediatric patients in at-home sessions, broadening the potential applicability of tremor entrainment therapy.

This study was not designed to evaluate efficacy of the Tremor Retrainer application, but all three subjects noted at least minimal improvement in tremor severity on a patient-rated CGI-I scale. It is possible this change, or a part thereof, may be attributable to a placebo effect. Patients and guardians answered this question differently, which could reflect some difficulty in redirecting attention from symptoms and separating the tremor from other functional neurological symptoms [7,8].

Our data are preliminary, and limited to three pediatric subjects. Ongoing software development is anticipated in response to needs of patients, clinical trial requirements, and software platforms. A future clinical trial will further evaluate feasibility and efficacy of the Tremor Retrainer application for the treatment of functional tremor.

## Conclusion

The Tremor Retrainer smartphone application can provide an auditory frequency determined by a user’s baseline tremor frequency and visual feedback on accuracy. Matching this frequency with flexion-extension movements at the wrist may help attenuate or eliminate the functional tremor by “hijacking” the abnormal native tremor frequency. Future studies regarding feasibility and efficacy of this biofeedback-based treatment of functional tremor are planned.

## Data Accessibility Statements

The Tremor Retrainer smartphone application and software code are available for review on request from the corresponding author (JG); the application code is not publicly available given its use in an ongoing clinical trial.
